# Metabolic diseases and pro- and prebiotics: Mechanistic insights

**DOI:** 10.1186/1743-7075-9-60

**Published:** 2012-06-19

**Authors:** Yukiko K Nakamura, Stanley T Omaye

**Affiliations:** 1 Department of Agriculture, Nutrition, and Veterinary Sciences, University of Nevada Reno, Reno, NV, 89557, USA

**Keywords:** Obesity, Type 2 diabetes, Inflammation, Lipopolysaccharides, Intestinal bacteria, Probiotics, Prebiotics

## Abstract

Metabolic diseases, such as obesity and type 2 diabetes, are world-wide health problems. The prevalence of metabolic diseases is associated with dynamic changes in dietary macronutrient intake during the past decades. Based on national statistics and from a public health viewpoint, traditional approaches, such as diet and physical activity, have been unsuccessful in decreasing the prevalence of metabolic diseases. Since the approaches strongly rely on individual’s behavior and motivation, novel science-based strategies should be considered for prevention and therapy for the diseases. Metabolism and immune system are linked. Both overnutrition and infection result in inflammation through nutrient and pathogen sensing systems which recognize compounds with structural similarities. Dietary macronutrients (fats and sugars) can induce inflammation through activation of an innate immune receptor, Toll-like receptor 4 (TLR4). Long-term intake of diets high in fats and meats appear to induce chronic systemic low-grade inflammation, endotoxicity, and metabolic diseases. Recent investigations support the idea of the involvement of intestinal bacteria in host metabolism and preventative and therapeutic potentials of probiotic and prebiotic interventions for metabolic diseases. Specific intestinal bacteria seem to serve as lipopolysaccharide (LPS) sources through LPS and/or bacterial translocation into the circulation due to a vulnerable microbial barrier and increased intestinal permeability and to play a role in systemic inflammation and progression of metabolic diseases. This review focuses on mechanistic links between metabolic diseases (mainly obesity and type 2 diabetes), chronic systemic low-grade inflammation, intestinal environment, and nutrition and prospective views of probiotic and prebiotic interventions for the diseases.

## Background

Obesity and overweight are world-wide health problems afflicting various populations, regardless of age, gender, and ethnicity. Obesity and overweight are abnormal conditions with excess fat accumulation, and are often determined by body mass index (BMI); BMI ≥25 is overweight while BMI ≥ 30 is obesity. According to data from the National Health and Nutrition Examination Survey (NHANES) 2007–2008, 68.0% of adults are overweight or obese, while16.9% of children and adolescents are obese in the U.S. [1,2]. Although no significant increase in obesity was observed among U.S. adults over the past ten years, the prevalence of obesity (33.8%) remains high. In contrast, the prevalence of obesity among U.S. children and adolescents has significantly increased over the past few decades [2,3], alerting health agencies to the serious health-related issue which has become wide-spread in the young population. Obesity and overweight have been thought to be a consequence of energy imbalance consisting of over-consumption of energy-dense foods (i.e., high-fat and high-sugar diets) and physical inactivity. Data from NHANES 1971–2000 reveals that average energy intake has significantly increased from 2450 kcal to 2618 kcal for U.S. men and from 1542 kcal to 1877 kcal for U.S. women during the past three decades [4]. The increase in the average energy intake is attributed to increased intake of fats (in grams) and carbohydrates, primarily as consumption of beverages providing simple sugars [5]. Similarly, the averages of daily energy intake among U.S. children (3–19 years old) were 2280 kcal (boys) and 1793 kcal (girls), and the energy intake was positively associated with BMI percentile from the age of six [6].

Diabetes is characterized by impaired secretion and/or activity of insulin and increased levels of blood glucose. The estimated case number of type 2 diabetes world-wide in 2000 is 171 million, translating into 2.8% of total population [7]. Wild et. al. predict a continuous increased trend of type 2 diabetes along with a stable prevalence of obesity; for example, an estimated prevalence of type 2 diabetes is 4.4% in 2030. Diabetes is one of the main causes of death in the U.S. According to CDC, 25.8 million of U.S. people (including 7 million of undiagnosed diabetes) have diabetes, accounting for 8.3% of total population. In 2010, an additional 79 million of U.S. adults are estimated to have prediabetes in which blood glucose levels exceed the normal range. Total costs of diabetes in 2007 were $174 billion, including $116 billion of direct medical costs and $58 billion of indirect costs (e.g., work loss, premature death, related disability) [8]. The epidemic of type 2 diabetes in the U.S. is associated with changes in diet, particularly an increase in intake of refined carbohydrates, including beverages, and decrease in intake of fiber during the past century [9].

Subsequently based on such national statistics, we could conclude that traditional approaches, such as diet and physical activity, have been unsuccessful in decreasing the prevalence of obesity and related diseases. Because these approaches strongly rely on individual’s behavior and motivation, we should consider other novel science-based strategies for prevention and therapy of metabolic diseases, particularly for weight loss.

### Metabolic disease-associated inflammation and endotoxiaemia

Obesity and overnutrition (long-term intake of high fat/sugar diets) are positively associated with chronic systemic low-grade inflammation, oxidative stress, and risks of other metabolic diseases, such as type 2 diabetes, cardiovascular disease, and some types of cancer [10-12].

Intestinal inflammation is associated with high fat diets and is considered as an early event in obesity and insulin resistance [13,14]. Diets high in meat products contain lipopolysaccharides (LPS) which are inducers of Toll-like receptor 4 (TLR4) [15]. TLR4 is an innate immune receptor which is localized on the surface of various cells. LPS are found in the outer membrane of gram-negative bacteria (e.g., E. coli.) and serve as endotoxins. Toxicity is associated with the lipid portions of LPS, lipid A, in which all of fatty acids are saturated, while immunogenicity is caused by their polysaccharide portions which contain O-antigens [16]. Released lipid A initiates a series of immune responses in the circulation after bacteria are lysed by host immune system. The immune system includes LPS-detoxifying properties through the intestinal alkaline phosphatase (IAP)[17]. However, dietary LPS can trigger immune responses to the hosts due to their resistance to temperatures and low pH, even though their carriers (e.g., meat products, processed foods) are edible [15,18]. LPS-induced TLR4 activation leads to inflammation by secreting pro-inflammatory cytokines and chemokines. Dietary fats increase absorption/plasma concentrations of LPS and TLR4 expression of mononuclear cells in normal humans [19]. Increases in the expression and activity of TLR4 and endotoxicity were observed in the monocytes of patients with metabolic syndrome [20]. LPS are internalized and transported by chylomicrons along with dietary fats in the circulation. These processes contribute to diet-induced systemic (either acute or chronic) inflammation and eventually the development of metabolic diseases, such as obesity, type 2 diabetes, and cardiovascular diseases, which are associated with chronic systemic low-grade inflammation [13,21]. A positive association was found between serum LPS activity and biomarkers of metabolic syndrome (e.g., triglyceride levels, insulin resistance, chronic inflammation) in type 1 diabetic patients [22]. Also, plasma levels of LPS-binding protein (LBP) were high in both obese-prone and high-fat-diet fed mice, compared to controls fed standard diets [23]. LBP is an acute phase protein to transfer LPS to CD14 on the cell surface and a biomarker of metabolic endotoxiaemia. In obese humans, compared to normal-weight controls, LBP levels were high and positively associated with biomarkers of metabolic syndrome and type 2 diabetes [24]. Thus, LPS-induced inflammation and endotoxiaemia are closely linked to obesity and type 2 diabetes.

### Dietary macronutrient-induced inflammation

Metabolism and immune system are linked. Undernutrition results in immunosuppression or susceptibility to infection, while overnutrition (i.e., obesity) leads to immunoactivation or susceptibility to inflammatory diseases, such as diabetes. Overnutrition (long-term intake of high fat/sugar diets) and infection lead to chronic and acute inflammation through nutrient and pathogen sensing systems, respectively [25].

Dietary macronutrients can act as inducers of TLR4 activation [26-29]. Lipids are non-immunogenic and can be haptens. The nutrient and pathogen sensing systems are likely to recognize compounds with structural similarities (e.g., saturated fatty acids in diets, lipid A derived from pathogens) and subsequently lead to the same consequence, inflammation (i.e., chronic or acute). Obesity-associated chronic inflammation could be characterized by a continuous activation of the innate immune system [30] and induced by overnutrition. LPS-induced TLR4 activation seems to be involved in either acute (i.e., infection) or chronic (i.e., overrnutrition) inflammation. Saturated fatty acids, not unsaturated fatty acids, induce inflammatory responses through TLR4 activation [28]. Eicosapentaenoic acid (ω-3, C20:5) and decosahexaenoic acid (ω-3, C22:6) are well-studied polyunsaturated fatty acids, which have exhibited anti-inflammatory and anti-diabetic properties mainly in animal models [31-34]. These ω-3 fatty acids down-regulate inflammation and adiposity by up-regulating adiponection via peroxisome proliferator-activated receptor gamma (PPARγ) activation and β-oxidation via PPARγ activation, respectively [34]. Similarly, lipid A containing unsaturated fatty acids are non-toxic and can serve as an antagonist against endotoxins [35,36].

Among unsaturated fatty acids, conjugated linoleic acid (CLA) is a group of geometric and positional isomers of linoleic acid (ω-6, C18:2), and is found mainly in dairy products and synthesized in the rumen as an intermediate by gram-negative bacteria, Butyrivibrio fibrisolvens, during the biohydrogenation of linoleic acid to stearic acid [37]. Some Lactobacillus species also produce significant amounts of CLA [38] (see the section of “potentials of probiotics and prebiotics” below). CLA is a possible PPARγ agonist [39], and has been shown to have health promoting properties such as anti-oxidant, anti-inflammatory, anti-carcinogenic, anti-atherogenic, and anti-obesity effects [40-44]. CLA exhibited dose-dependent differential effects (i.e., prooxidant or antioxidant, cytotoxic) associated with redox-sensitive transcription factors PPARγ and NF-B in human endothelial cells [44,45]. These transcription factors seem to modulate oxidative stress and inflammation in a coordinated fashion, depending on micro-environmental factors [46]. Anti-obesity effects of CLA in humans are supported by two meta-analyses of Whigham et al. [47,48]. Kennedy et al. [40] conclude in their review that anti-obesogenic effects of CLA are due to: 1) reduction of energy intake with suppressed appetite, 2) induction of energy expenditure in white adipose tissue, muscles, and liver tissue, 3) reduction of lipogenesis or adipogenesis, 4) induction of lipolysis, and 5) induction of adipocyte apoptosis.

In addition to dietary fats, dietary carbohydrates appear to be involved in inflammation through TLR4 activation. High glucose treatment induces TLR4 expression in human monocytes [26], while insulin reduces LPS-induced TLR4 activation and oxidative stress [49,50]. Free saturated fatty acids exacerbate the expression and activity of TLR4 which is induced by high glucose in human monocytes along with increases in superoxide generation, NF-κB activity, and pro-inflammatory factors [51]. A decrease in the levels of the insulin-sensitive glucose transporter (GLUT4) is a characteristic of type 2 diabetes and insulin resistance. Insulin resistance results from down-regulation of GLUT4 and glucose transport selectively in adipose tissue [52]. Reduced levels of GLUT4 found in type 2 diabetes appear to be in part due to GLUT4 expression suppressed by free fatty acids through PPARγ [53]. PPARγ is known to be an adipogenic factor which triggers adipocyte differentiation. However, PPARγ agonists (e.g., thiazolidinediones) cause PPARγ protein to dissociate from the GLUT4 gene promoter and improve insulin resistance, by blocking suppression of GLUT4 mediated by fatty acids (i.e., arachidonic acid) and/or TLR4 agonists (i.e., LPS) and inducing GLUT4 expression via PPARγ activation [53-55]. In summary, an association is suggested between dietary macronutrients, inflammation through TLR4 activation, redox-sensitive transcription pathways, and metabolic diseases.

### Involvement of intestinal bacteria in the pathogenesis of metabolic diseases

Because the gut is exposed to foreign antigens in foods, the gut associated lymphoid tissue (GALT) has evolved mechanisms to avoid strong immune responses to food antigens and to protect against pathogenic organisms derived from foods. Human gut bacteria consist of approximately 10^14^ colony-forming-unit (cfu)/mL and 500 to1000 species and live in symbiosis with their host [56]. Bacteroidetes and Firmicutes are dominant (>90% of total microbial population) in the normal mouse and human intestines [57]. Bacteroidetes and Firmicutes play a role in nutrient absorption, mucosal barrier fortification, xenobiotic metabolism, angiogenesis, and postnatal intestinal maturation [57-59]. The population of these bacteria is controlled by diets (i.e., high fat) [60], and is crucial in development of obesity and diabetes [57,61,62]. The relative abundance of Bacteroidetes was higher in diabetic-prone rats with the development of diabetes than those without the development [61]. A decrease in Bacteroidetes and increase in Firmicutes were observed in obese humans and mice [57,60]. The changes in these phyla depend on diets (i.e. high fat) regardless of phenotypes (i.e., lean, obese-prone, obese-resistant) [60,62]. In addition, differences in types of immune cells present in the adipose tissue of obese and lean mice may be a consequence of differences in microbial composition [63].

Recent findings that support the idea of the involvement of intestinal bacteria in the development of obesity and diabetes include: 1) the resistance to high-fat diet-induced obesity in germ-free mice [64], 2) antibiotic-induced reduction of plasma LPS levels in obese mice fed a high-fat diet [65], and 3) delayed onset and development of type 1 diabetes by use of antibiotics in a diabetes-prone rat model [61]. Also, chronic intake of high-fat diets modulate intestinal inflammation through alteration of intestinal environment, such as intestinal permeability, microbial composition, and LPS-detoxifying ability of the intestinal alkaline phosphatase (IAP: an LPS detoxifying enzyme) activity [62,66]. Certain types of intestinal bacteria appear to serve as sources of LPS through the translocation of LPS and/or bacteria [67,68]leading to chronic low-grade inflammation locally and/or systemically. Furthermore, these studies reveal that energy imbalance, which results from high calorie diet and physical inactivity, is not the only factor to attribute to development of obesity and metabolic diseases.

Thus, specific intestinal bacteria may provide LPS to induce low-grade inflammation locally and/or systemically through the translocation of intestinal bacteria and/or their products, and play a role in host metabolism. Since the mechanistic links are suggested mainly in animal models, further investigation is needed to determine mechanisms in humans.

### Potentials of probiotics and prebiotics

Probiotics, such as Lactobacilli and Bifidobacteria, are defined as “live microbial feed supplements which beneficially affect the host animal by improving its intestinal microbial balance”[69]. Prebiotics are non-digestible, fermentable carbohydrates and fibers, such as inulin-type frucans and galacto-oligosaccharides, which exhibit health promoting properties to host through selective stimulation of growth and/or activities of a limited number of bacteria (i.e., probiotics). Since probiotic- and prebiotic- induced health promoting effects are likely to be attributed to their ability to antagonize pathogenic bacteria and to modulate host immune responses [70,71], earlier studies mainly focused on the relationship between probiotics/prebiotics and immune diseases (e.g., atopic disease, asthma) and infant nutrition [72-74]. In 2007, Gordon and his colleagues first reported the involvement of intestinal microbes in obesogenesis [64]. Afterward, some investigators have documented that altered intestinal environment (i.e., microbial composition, intestinal permeability, LPS-detoxifying ability) could contribute to adiposity and insulin resistance [61,62,64-66,75], as described in the previous section. Several investigations have been done on the effects of probiotics and prebiotics for obesity and diabetes [68,76-79].

Investigation of probiotics (e.g., Lactobacilli, Bifidobacteria) and prebiotics (e.g., inulin, oligofructose) have lead to the suggestion that these supplements could have preventative and therapeutic potentials for immune diseases, exhibiting bacteria-specific immunomodulatory properties [71,80-82]. First, probiotics, such as Lactobacillus casei, Lactobacillus paracasei, Lactobacillus acidophilus, and Bifidobacterium animalis, can survive and grow at 20-40% of the estimated survival rate in the GI tracts after their oral administration and increase their population in the intestines [83-85]. Low abundance of Lactobacilli and Bifidobacteria is associated with development of type 1 diabetes [86]. Similarly, high levels of Lactobacillus casei/paracasei and Lactobacillus plantarum in human gut are negatively associated with obesity, but high levels of Lactobacillus reuteri are positively associated [68,87]. In addition, Lactobacillus species were decreased in the distal esophagus of rats fed a high fat diet, compared to those fed the standard diet [88]. Yogurt containing Lactobacillus acidophilus exhibits anti-cholesterolemic properties in mature boars [89]. Anti-inflammatory effects of probiotics, Lactobacillus casei, are negatively associated with NF-кB p50/p65 activation [90], which is induced by LPS through TLR4 activation, and positively associated with PPARγ activation [91].

Anti-obesity effects were observed in C57BL/6 J diet-induced obese mice supplemented with 1 × 10^9^ colony-forming unit (cfu) of Lactobacillus rhamnosus PL60 which can produce CLA [79]. CLA are also produced from linoleic acid by Lactobacillus acidophilus, Lactobacillus plantarum, Lactobacillus paracasei, and Lactobacillus casei in vitro and in vivo (i.e., mice) [38,92]. CLA-induced adverse effects, such as increased insulin resistance and inflammation, have been observed mainly by use of single purified CLA isomer (in particular the trans-10, cis-12 CLA isomer) through NF-кB p50/p65 activation [93-95], while CLA producing Lactobacillus species (e.g., L. casei, L. plantarum) exhibited anti- inflammatory effects along with increased PPARγ expression [92]. CLA produced by probiotics in vivo appears to remain within the intestinal lumen and serve as a PPARγ agonist locally, whereas orally supplemented CLA seems to be absorbed and affect systemically [96].

As for prebiotics, Parnell and Reimer demonstrated that obese and lean rats fed a diet containing inulin and oligofructose (0, 10, 20% w/w) increased anorexigenic peptide levels and probiotic population and decreased glucagon levels in a dose-dependent manner. However, no significant changes in body weight or blood insulin/glucose were observed between rats fed with or without prebiotics [77]. In contrast, prebiotic treatments (0.3 g/mouse/day) exhibited anti-obesity, anti-diabetic, antioxidant, and anti-inflammatory effects in obese mice and altered intestinal microbial composition [76].

Exosaccharides or extracelluar polysaccharides (EPS) consisting of sugar residues are bound to the cell surface of gram-positive/negative bacteria, or secreted as soluble/insoluble polymers [97]. Probiotic-derived EPS are counterparts of LPS produced by pathogenic bacteria, and antagonize the bacteria and endotoxins [98,99]. EPS is heat resistant with its degradation temperature of 260 °C [100], similar to LPS. Supplementation of EPS isolated from Bifidobacterium results in immunomodulatory effects (5 g/mL of EPS) in macrophages and anti-microbial effects against pathogenic bacteria (80 g/mL of EPS) [101]. Oral administration of EPS (100 mg/kg body weight/day of Lactobacillus kefiranofaciens for 2 to 7 days) effectively induces systemic immunity through cytokines released into the circulating blood of mice [102]. Hypoglycemic effects of EPS derived from mushrooms (Tremella fuciformis and Phellinus baumii) were observed in obese mice, and the effects were associated with increased PPARγ expression [103]. Lactobacillus species-derived EPS (kefiran) prevents the onset and development of atherosclerosis in hypercholesterolemic rabbits fed diet with 1% w/w kefiran through anti-inflammatory and antioxidant properties [104]. EPS isolated from Lactobacillus paracasei exhibits immunomodulatory and antioxidant properties in a dose-dependent manner [105].

Therefore, one is lead to speculate that health promoting properties of probiotics could be related to PPARγ activation through their products by antagonizing intestinal pro-inflammatory bacteria, blocking the NF-кB p50/p65 activation which is induced by LPS derived from the bacteria, and modulating intestinal environment/microbial composition. Clinical trials of pro-/prebiotic intervention are needed, as the speculation is attributed to mainly results from animal studies.

### Conclusions: Prospective insights

**Figure 1:**
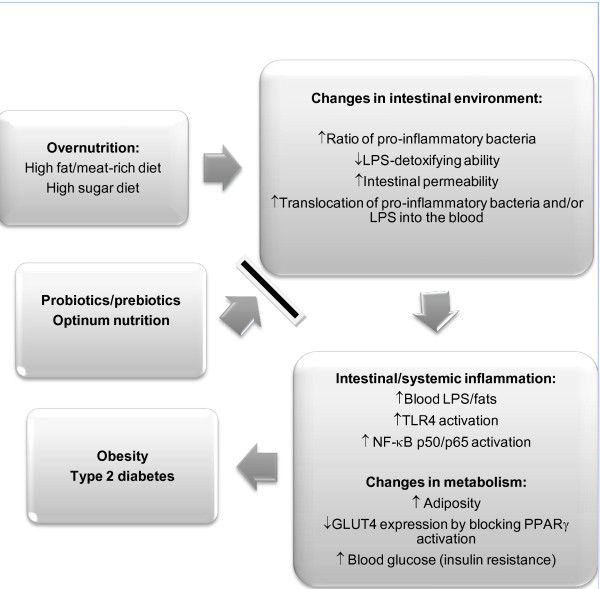
**Mechanistic links of metabolic diseases.** Overnutrition (chronic intake of high fat/sugar diets) may modulate intestinal environment, and subsequently may lead to chronic low-grade inflammation locally and systemically and alter metabolism.

**Figure 2:**
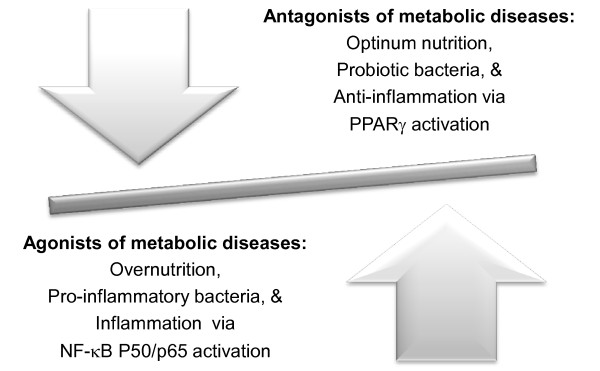
**Relationships at microbial and molecular levels in metabolic diseases.** Nutrition status, classes/species of intestinal bacteria, and signaling pathways could be determinants in the development of metabolic diseases and be expressed as agonists (inducers) or antagonists (suppressors) of the diseases.

Mechanistic links of metabolic diseases are shown in Figure 1. Optimum nutrition includes long-term intake of diets rich in unrefined carbohydrates (or fibers/prebiotics), while overnutrition refers to long-term intake of diets high in fats/meat (particularly saturated fats) and refined carbohydrates (or simple sugars). Chronic intake of diets high in fats and sugars may alter intestinal environment, including microbial composition and mucosal structure/functions, and result in a vulnerable microbial barrier and increased permeability of the intestines or leaky gut [67]. These changes allow intestinal bacteria and/or LPS to move into the circulation, and eventually lead to chronic systemic low-grade inflammation associated with metabolic diseases [67,106]. Nutrition status, classes/species of intestinal bacteria, and signaling pathways could be classified as agonists (or inducers) or antagonists (or suppressors) of metabolic diseases. Overnutrition, increased ratio of pro-inflammatory bacteria to total intestinal bacteria, and NF-кB p50/p65 activation could serve as agonists of metabolic diseases, whereas optimum nutrition, optimum ratio of probiotic bacteria (e.g., Lactobacilli, Bifidobacteria), and PPARγ activation could act as antagonists of the diseases (Figure 2). The antagonists may be able to alleviate or reverse processes induced by the agonists. As previously described, classes/species and/or ratio of pro-inflammatory bacteria remain to be determined because the results of existing investigations were inconclusive. Further research are needed to clarify classes/species or ratio of the bacteria to total bacterial population.

The control of intestinal microbial composition by use of probiotics and prebiotics is likely to impact the development of metabolic diseases through modulation of immune responses/inflammation and metabolism. Supplementation of probiotics and prebiotics may delay and/or reverse the progression of metabolic diseases. Additional preclinical and clinical investigations are warranted to determine the relationship between metabolism and immune system and the efficacy of probiotics and prebiotics as preventative and therapeutic means for metabolic diseases.

## Abbreviations

LPS, Lipopolysaccharides; TLR, Toll-like receptors; GLUT4, Glucose transporter 4; EPS, Extracellular polysaccharides; CLA, Conjugated linoleic acid; PPARγ, Peroxisome proliferator-activated receptor gamma; NF-кB, Nuclear factor kappa B.

## Competing interests

The authors declare that they have no competing interests.

## Authors’ contributions

Both authors participated in the preparation of the manuscript, and read and approved the final manuscript.
